# Recent Advances in Microneedle-Based Sensors for Sampling, Diagnosis and Monitoring of Chronic Diseases

**DOI:** 10.3390/bios11090296

**Published:** 2021-08-25

**Authors:** Özgecan Erdem, Ismail Eş, Garbis Atam Akceoglu, Yeşeren Saylan, Fatih Inci

**Affiliations:** 1 UNAM—National Nanotechnology Research Center, Bilkent University, Ankara 06800, Turkey; ozgecan@unam.bilkent.edu.tr (Ö.E.); ismail@unam.bilkent.edu.tr (I.E.); garbisakceoglu@gmail.com (G.A.A.); 2 Department of Chemistry, Hacettepe University, Ankara 06800, Turkey; yeseren@hacettepe.edu.tr; 3 Institute of Materials Science and Nanotechnology, Bilkent University, Ankara 06800, Turkey

**Keywords:** chronic disease, biomarkers, detection methods, microneedles, biosensor

## Abstract

Chronic diseases (CDs) are noncommunicable illnesses with long-term symptoms accounting for ~70% of all deaths worldwide. For the diagnosis and prognosis of CDs, accurate biomarker detection is essential. Currently, the detection of CD-associated biomarkers is employed through complex platforms with certain limitations in their applicability and performance. There is hence unmet need to present innovative strategies that are applicable to the point-of-care (PoC) settings, and also, provide the precise detection of biomarkers. On the other hand, especially at PoC settings, microneedle (MN) technology, which comprises micron-size needles arranged on a miniature patch, has risen as a revolutionary approach in biosensing strategies, opening novel horizons to improve the existing PoC devices. Various MN-based platforms have been manufactured for distinctive purposes employing several techniques and materials. The development of MN-based biosensors for real-time monitoring of CD-associated biomarkers has garnered huge attention in recent years. Herein, we summarize basic concepts of MNs, including microfabrication techniques, design parameters, and their mechanism of action as a biosensing platform for CD diagnosis. Moreover, recent advances in the use of MNs for CD diagnosis are introduced and finally relevant clinical trials carried out using MNs as biosensing devices are highlighted. This review aims to address the potential use of MNs in CD diagnosis.

## 1. Introduction

From a medical perspective, chronic diseases (CDs) are noncommunicable illnesses with persistent symptoms requiring at least one year of continuous medical treatment [1]. According to the Fact Sheet reported by World Health Organization (WHO) on 13 April 2021, these diseases are responsible for ~70% of all global deaths annually [2]. Among all CDs; cancer, cardiovascular, and respiratory diseases are the leading ones accounting for most deaths [2]. In addition to financial burden, they cause tremendous psychological suffering for those who are diagnosed. Therefore, early diagnosis and continuous disease monitoring in combination with timely treatments are critical measures to improve the quality of patients’ lives and prevent or decelerate disease progression [3].

A valid measurement of biomarkers is vital for the diagnosis and prognosis of CDs [4,5,6]. Currently-available health technologies for CD biomarker detection involve the use of sophisticated techniques, such as mass spectrometry [7], chromatography [8], ELISA [9], and PCR [10]. Although these techniques are considered gold standards for clinical diagnosis due to their high sensitivity and specificity, they are the part of centralized testing [11], where samples are collected from the patient in a hospital, and then, delivered to another laboratory for analysis [12]. Moreover, clinical practices of these techniques face a number of limitations as they involve laborious, time-consuming, and costly procedures. In such a scenario, the test result is typically not available to the patient until the next day. Therefore, decentralized testing is highly required as a point-of-care (PoC) concept for the rapid and accurate detection of such diseases [5,13,14] and any conditions requiring further downstream analyses [15,16,17,18]. Recently, considerable attempts have been made to manufacture PoC platforms for CD diagnostic and monitoring. For instance, according to the guidelines reported by the WHO, a PoC device should meet the ASSURED criteria (affordable, sensitive, specific, user-friendly, rapid and robust, equipment-free and deliverable to end-users) [19,20,21]. Nowadays, with advances in mobile health (m-health) technologies and communication systems, the authorities suggest two additional points (R: real-time connectivity; E: ease of specimen collection and environmental friendliness), turning it into a new acronym of REASSURED [22]. Moreover, several PoC devices have been commercialized for the diagnosis and monitoring of CDs. Considering new trends in this domain, microneedle (MN) technology has emerged as a game-changer in the biosensor and drug delivery fields for the purpose of PoC technologies.

Briefly, an MN-based sensing device consists of micron-sized arrays that are arranged on a miniature patch in a specific order. MNs are currently manufactured as solid, hollow, coated, and dissolving modes with a length and width of 150–1500 μm and 50–250 μm, respectively [23]. MN-based sensing systems present remarkable advantages over the other PoC devices as they are rapid, easy-to-use, and reliable devices with pain-free and minimally invasive properties. Although MNs were first conceptualized in the 1970s (US Patent number US3964482A), they were not broadly investigated due to the lack of microfabrication techniques until the late 1990s [24]. Since then, numerous MN-based platforms have been manufactured for different purposes using a variety of materials, such as polymers [25], glass [26], ceramic [27], and metal [28] with different shapes (e.g., conical, pyramidal, etc.). Fabrication techniques, such as dry or wet etching, ion etching, laser ablation, photolithography, 3D printing, and micro-molding techniques were shown to be highly effective in designing these sensing platforms [23].

In recent years, there has been enormous attention on the development of MN-based biosensors for the real-time monitoring of CDs (e.g., cancer, diabetes, and cystic fibrosis) by puncturing the epithelial tissue (e.g., stratum corneum or intestinal epithelium) for the analysis of biomarkers [29]. Biofluids, such as interstitial fluid and blood, are rich sources of biomarkers [30], where MN-based biosensors can be applied for transdermal sampling and continuous electrical read-out of signals. MNs can be an active component of biosensors or have the function of sample collections to further deliver it to the biosensor for the read-out of the signal [29]. For both cases, MNs alone may not present satisfying results in terms of biomarker monitoring, and hence, coating of MN surface through materials, such as gold (Au) or nanocomposite films [31] can be an effective strategy to enhance the detection performance. Moreover, the coating material can be functionalized additionally with biological molecules, such as aptamers or specific peptides, to increase the precision of electrochemical sensor for more efficient binding to the molecules excessively secreted in the case of CDs [32]. Interestingly, the design of MN patch is also an essential part of the MN-based biosensor system. The design parameters such as geometry, size, mechanical properties, and materials should be carefully considered for further applications.

There is an urgent need for more reliable biosensing devices to provide timely feedback to patients diagnosed with CDs. Taken altogether, design parameters such as geometrical design, materials, fabrication, and coating methods of MNs should be further investigated to develop a more accurate MN-based sensing system for CD detection. Recent reviews broadly focus on the use of MNs for drug delivery purposes [33,34,35]. There is a limited number of review on MN-based platforms as biosensing devices [29,36]. Different from other reviews, this review focuses on the state-of-the-art progresses of the MNs-based sensor systems to bridge the gaps between MNs and biosensors for the detection of CDs. In particular, the basic concepts of MNs and their fabrication techniques are briefly introduced then their integration with sensors to detect CD is critically analyzed and the strategies to increase their accuracy are discussed. Finally, their applications in clinical trials are highlighted.

## 2. Materials and Methods for Microneedle Fabrication

MN fabrication techniques and material composition may vary depending on the needs and purpose of their applications (Table 1). By using different techniques, complex structures such as arrowhead, and angled MNs orientated on surfaces such as honeycomb pattern, can be produced [37,38]. Parameters such as aspect ratio, height, base diameter, patch area, and inter-needle spacing should be carefully considered in designing and fabricating MNs with different geometric structures. Additionally, high uniformity is required for high-throughput fabrication [39]. The main reason for this concern is because skin shows a certain amount of tensile strength against MNs, and the developed MNs need to possess certain mechanical strength without breaking or cracking for effective skin penetration. Therefore, choosing an appropriate fabrication method is essential, and it highly depends on the materials to be used. For example, microelectromechanical systems (MEMS) are suitable for metals contrary to soft and degradable materials [40]. Until today, MNs have been manufactured utilizing inorganic materials such as silicon, glass, and ceramics, as well as metals such as aluminum and titanium by different methods [35,41,42,43]. With the increasing importance of biodegradable and soluble MNs, polymers and hydrogels have attracted the attention of researchers and new manufacturing techniques, such as 3D printing have been proposed [35,44].

MNs are major player in removing the limitations observed in conventional transdermal applications of the diagnostics or patient monitoring [33,45]. A number of MN designs and application strategies have been developed, however, it is very critical to create the optimum MN design according to the desired application from among the different options. MNs have been applied to detect biomarkers such as nucleic acid, proteins, small molecules, and even cells from interstitial skin fluid (ISF) for early diagnosis and real-time monitoring. In this sense, being able to detect the ISF biomarkers has many advantages over blood, saliva or urine samples. The MNs which can detect biomarkers, majorly use two different working mechanisms for biomarker detection. The first method is to absorb samples from ISF, and then extract the samples from MNs and analyze them with other instruments [46,47,48]. The second method is to place all detection processes and stages that will recognize all relevant biomarkers in all-in-one MNs. Specific probes that will recognize target biomarkers are coated or encapsulated on MNs [49,50]. These detection stages inside MN array are connected to a reservoir to absorb the ISF fluids. This reservoir connection can be a microfluidic chip or a paper-based chip that can provide detection results, as well as sample collection [48,51]. Despite all their benefits, reservoir structures would cause difficulties in application and production stages, for this purpose, it is desired to produce much facile systems instead of reservoir structures. Hence, researchers have presented a MN with high swelling capacity materials [47]. Such MNs are able to quickly absorb enough ISF; then biomarkers are extracted from the MNs using elution, centrifugation or other milder methods.

MN-based platforms usually consist of two parts: (1) MN array that opens micropores in the skin through which biomarkers can be collected; (2) MN patch backing where the MNs are orientated on a surface. Patch backing can be fabricated together with the MN array or separately to further adhered to the arrays [52]. Patch backing materials can be made of highly viscous or hydrophobic materials, so the diffusion or movement of the drugs or polymers attached to the surface of MN can be reduced. The patch backing can be separated from MN array by a tiny air bubble or a porous layer, and thereby, the patch backing can be easily detached from MN arrays. Designing such a patch backing is essential to provide a barrier to drug migration. However, the material selection to manufacture patch backing can vary depending on the hydrophilic/hydrophobic nature of the drug or the type of polymer coated to the surface of the MN. It was also shown that using materials with hydrophobic nature (i.e., polyvinyl alcohol (PVA)/sucrose) can reduce the amount of residue left on the backing material [53].

The MNs are divided into four main groups depending on their structure and usage strategies: (i) solid MNs that pre-treat the surface of the skin [54], (ii) coated MNs containing water-soluble biomolecules on their tips for capturing markers [55], (iii) dissolving MNs [56], and (iv) hollow MNs that contains a reservoir for the movement of the sample [57]. Although hypodermic needles have been reported for causing pain and psychological stress on patients for about 170 years, they still play the core role in the administration of drugs and bioactive molecules, and therefore, all types of MNs are always compared to hypodermic MNs, which are considered the gold standard [58].

**Table 1:** Fabrication methods and materials of different microneedles.

Type of MNs	Material Used for Fabrication	Fabrication Method
Solid	Silicon Titanium Stainless steel Glass Ceramics Nickel-iron	Laser ablation Laser cutting Casting Electroplating Lithography Wet and dry etching methods Metal injection molding Micromolding Two photon polymerization
Coated	Metal or silicon materials for solid base	Micromolding Dip coating Spray coating Layer-by-layer manufacturing
Dissolving	Carbohydrates PVA, PVP, PLA, PLGA Sodium carboxymethyl cellulose	Mold based techniques Drawing lithography UV assisted fabrication Heat Droplet air blowing (DAB) Fused deposition modeling (3DP) Atomized spray process
Hollow	Silicon Metal Polymer Ceramic Glass	MEMS Deep reactive ion etching Photolithographic Micromachining Pipette technique Deep x-ray lithography

### 2.1. Solid Microneedles

Solid MNs can be designed in different ways to form microchannels on the skin surface across the epidermis and dermis. These microchannels allow drugs or bioactive compounds to be delivered to the body through the dermis layer. These compounds are then transported to the desired places on the body by systemic circulation [59]. Thanks to advanced technologies and materials science, solid MNs can be produced according to the projected lengths and shapes with the help of different materials such as silicon, titanium, stainless steel, ceramics, glass and nickel-iron. Bal et al., for instance, have reported that various shapes and speed of applications are affecting the depth and width of the pores [60,61,62,63,64]. Compared to the other MNs, solid MNs are very easy to manufacture and use, as well as they can even be reused. Today, the most preferred materials for MN production are titanium, stainless steel, and ceramics [64,65]. They are fabricated on flat surfaces such as spike plates, and then, directly cut by laser ablation or laser cutting. Besides these techniques, they can be made from a master mold by casting, electroplating, lithography, as well as wet and dry etching methods [66,67]. Metal injection molding technology was used by Li et al. in order to create titanium porous MN arrays (TPMA), which exhibited 27 times higher calcein concentration across intact skin [68]. On the other hand, the micromolding technique is the most widely employed method for the production of ceramic MNs, and it is a low-cost technique compared to other methods, as well as it offers the advantage of potential up-scaling technology. In this method, ceramic MNs were produced by casting alumina slurry into PDMS MN molds, and then, sinter.

In another study, Ormocer^®^ MNs were produced using two-photon polymerization technique at different aspect ratios, and these MNs were able to penetrate inside porcine adipose tissue without cracking [69]. Ceramic MNs have also been used in vaccine delivery, and it has been observed that the effect of the vaccine was increased by reaching the dendritic cells of the skin, where T and B immune cells are locating [70]. Even though today other materials are the most preferred materials, the first solid MN samples were produced from silicon by using the lithography, and wet and dry etching method―costly fabrication processes [24,71,72]. Later on, super short MNs were produced in 30% KOH solution at 80 °C by using the wet etching method to reduce the production cost [73,74]. Silicone material allows to create MNs at various geometry very easily. For instance, Narayanan et al. fabricated silicone MNs using tetramethylammonium hydroxide (TMAH) solution at different concentrations, temperatures, time, and the rate of etching in order to create sharper MNs with a higher aspect ratio. Similarly, another etching technique was applied on silicon MNs by using isotropic potassium hydroxide for topical immunization with naked plasmid DNA. This work demonstrated that MN-assisted vaccinations caused less variable and stronger immune responses compared to hypodermic injections [75]. Besides etching methods, micromold fabrication is another method used for producing solid MNs and it has considerable advantages over etching and laser cutting strategies through the formation of exact replicas by a mold [40]. Additionally, these molds can be produced by 3D printing technology for solid MNs with a high aspect ratio [37].

### 2.2. Coated Microneedles

Coated MNs are based on solid MNs, and they are formed by coating drug solutions, macromolecules, small molecules, vaccines, or micron-size particles on their structures [76,77,78,79]. Moreover, the molecules, such as vitamin B, bovine serum albumin (BSA), and calcein, were also reported as coating agents in this type of MNs [80,81]. Coated MNs have two main functions stemming from “the coat and poke” principle. The first of these is to pierce the skin, and the second is to release the drug to the body through bolus feeding. In addition, compared to the other types of MNs, they can remain stable for a long time due to their solid base, which are usually made of metal or silicon materials in order to provide sufficient mechanical strength [82]. Another study has shown that they can remain stable for 6 months at room temperature after being stored under nitrogen atmosphere [83]. On the other hand, despite their long stable time, the coated MNs have a relatively small surface area for drug absorption (less than 1 mg per array containing hundreds of MNs) [84]. To increase drug loading capacity, the coated MNs of different shapes can be fabricated.

Conventional production techniques such as micromolding usually fail to change MN design quickly and easily due to the need for precise preparation processes and cumbersome master templates. Therefore, many different methods, such as dip coating, spray coating, and layer-by-layer manufacturing, have been tested in the production of coated MNs [79,81,85,86,87,88]. Among them, dip coating method is the most utilized one due to its efficiency and simplicity [89]. However, it is necessary to ensure that the MNs accurately enter the dipping solution for a more uniform coating [81]. Another method that allows us to load drugs into MNs is the spray coating method, and for instance, it was previously reported that more suspended lidocaine could be loaded into the polyethylene glycol matrix employing this method [90]. During the spraying process, drug loss should always be considered [79]. In the layer-by-layer method, MNs are repeatedly dipped or sprayed into high-density liquids to form coated MNs [91]. Additionally, electrostatic forces can be also used in the layer-by-layer method to enable solid MNs to be coated with structures possessing different surface charges. As an example, negatively charged LB-MSN-DT and positively charged N-trimethyl chitosan (TMC) were coated onto pH-sensitive MN using this technique. In vivo tests in mice showed that coated MNs induced stronger DT specific antibody responses compared to hollow MNs.

### 2.3. Dissolving Microneedles

Dissolving MNs mainly work on “the poke and release” principle. They have gained remarkable attention in recent years due to low-waste generation, affordable and facile production, self-administration, cold chain distribution, and higher patient compliance among the other MNs [45,92]. They can also be made from water-soluble, inert, biodegradable, or natural polymeric materials, such as carbohydrates [71,93]. Being producible from these materials allow them to quickly dissolve under the skin and safely deliver the pharmaceutical agents in a controlled and painless way, making them an excellent candidate for disease diagnosis and treatment [70,94]. In addition, owing to the recent technological developments in polymer chemistry, biocompatible, biodegradable, mechanically-resistant and polymer-based dissolving MNs with optical clarity can be fabricated [95,96,97,98].

Dissolving MNs can be produced at low or room temperatures using mold-based techniques such as investment casting or hot embossing. Commonly, PVA [99], poly (vinyl pyrrolidone) (PVP) [100,101] poly (D,L-lactic acid) (PLA) [102], poly (D,L-lactic-co-glycolic acid) (matrix materials such as PLGA) [103], carbohydrates [104], and sodium carboxymethyl cellulose [105] are employed, and they distribute the drugs to the body in a sustained or bolus manner depending on the material [106]. The fabrication of sugar glass-based MNs at low temperatures and molten maltose-based MNs at room temperature were previously carried out using mold-based techniques [97,107]. In another study, MNs were also dissolved in SU-8 photoresist molds using denser liquids, such as biocompatible amylopectin or carboxymethylcellulose [92]. These MNs could successfully release encapsulated molecules within their shafts and completely dissolved without any residue. Soluble MNs were also fabricated through a lithography method using maltose as a structural matrix [108]. This method is applied to the transformation of 2D dense polymer materials into 3D polymer structure using extensional deformation [109]. This technique was also employed using matrix material, such as dextran [110], carboxymethyl cellulose [111], dextrin [112], polyvinyl pyrrolidine [112], chondroitin sulphate [113], PVA [114], fibroin [115], poly (lactic-co-glycolic) acid [116], and sugars. The drawing lithography technique was modified by adding a metal base plate to fabricate hybrid electro MNs (HEMNs) [117]. After penetrating the skin, the electrode connected to the HEMNs facilitated cutaneous release from the encapsulated reservoir by generating electric field pulses following a poke and controlled release approach to transdermal delivery [117].

UV-assisted fabrication and heat are widely used in conventional MN production methods, yet this leads to a reduction in the drug loading capacity of MNs. To hurdle this problem, the droplet-born air blowing (DAB) method was developed [118], and basically, here the polymer droplet takes the shape of the MN by air blowing applied to it. This creates a gentler fabrication condition for drugs compared to UV or heat [119]. Briefly, the process initiates with the dispensing of solution on upper and lower plates. The two plates are kept in contact and gradually pulled apart, hence elongating the viscous solution, which is then exposed to blowing air under controlled conditions. The required shape is given during this drying step. In addition, the use of a single polymer ensures the control of the droplet size and concentration used per MN, and it provides a drug load opportunity without any drug losses [118]. Employing this method, for instance, MNs containing insulin have been successfully produced, and insulin was delivered to diabetic mice without damaging their skin [120].

On the other hand, micromachining methods are still not preferred because of their high production cost and time-consuming processes. However, Luzuriaga et al. have introduced a new method that they applied fused deposition modeling (3DP) and managed to quickly design and manufacture MNs at the desired density, length, and shape. Furthermore, by combining the 3DP method with the chemical etching method, the MN tips can be reduced to 1 mm in size [121].

Another method used in the production of dissolving MNs is the atomized spray process, which is normally used in the production of different biopharmaceutical products. This method was used for the first time in the production of MNs, and it allowed the production of soluble MNs from sugar or polymers (i.e., PVA, trehalose, and sodium alginate) [122]. Since dissolving MNs could be produced in one step, this method would be scaled up for mass production. In addition, it is both simple and suitable for the use of substances, such as protein vaccine, since it does not require vacuum, centrifugation, high pressure or high temperature. With this method, Tarbox et al. succeeded in producing dissolving MNs with 5% solids using silicon micro molds [123]. Dissolving MNs through different production techniques have showed that the geometric properties of the MN are more crucial in drug delivery than the fabrication methods. Park et al. have reported that the release kinetics of compounds are directly related to the geometric shape of the applied needle and drug localization within the MN array [124].

### 2.4. Hollow Microneedles

Hollow MNs have emerged as the conventional hypodermic needles decrease to micron sizes with the development of material technologies [125]. Compared to other MNs, substantial doses can be delivered to the body in a controlled manner, satisfactorily and easily through the dermal layer by pressure-driven flow [65,126,127]. The drug volume solution and rate of delivery may be at risk of fracture due to the compaction properties of the skin or the insufficient mechanical strength of the MN [61,128]. However, in recent studies, it has been shown that this risk can be eliminated by using hyaluronidase or partial retraction method [129,130]. In addition, Bodhale et al. designed a special MN, especially for the clogging problem. They used a tapering needle shaft instead of straight-walled MNs and placed the lumen hole on the side of the MN array [131,132]. Although hollow MNs are very difficult to produce due to their fragility, many methods have been tried to produce them. One of the methods is MEMS, allowing them to be produced in large quantities. Based on the MEMS techniques, Yu et al. produced cylindrical hollow MNs using deep reactive ion etching, photolithographic, and micromachining processes [133]. Another method is the pulling pipette technique, which is used to produce glass hollow MNs. In this technique, soft surface MNs are formed by heating the glass to high temperatures and pulling it with a micropipette puller [92,134]. With the deep X-ray lithography method, hollow MNs can be made from metal, polymer, ceramic, or glass materials with high precision. In short, channels and triangular columns are formed by coating polymer solutions on silicon wafers and applying vertical deep X-rays on these wafers. Through continuous adjustment and alignment of the X-ray mask and the substrate, the anticipated geometry of MNs is generated [104,135]. Although great strides have been made in hollow MN production techniques, such as digital hollow MN injection systems [136], vaccine studies have not conducted studies on the development of these MNs due to the complex and lengthy process of production [119,137,138,139]. On the contrary, micron-sized hypodermic needles attached to the syringe with a length of 1000–1500 m and 30–34 gauge of outer diameter are still used in vaccine studies [24]. Hollow MNs are used in different areas apart from vaccination studies. For example, Miller et al. have integrated such MNs into microfluidic chips and created a new type of transdermal sensor-based PoC device to be used in the diagnosis of CDs [98].

## 3. Applications of MNs in the Detection of CD Biomarkers

In recent years, different types of MN-based biosensors including electrochemical, optical, magnetic, and paper-based have been studied in the literature [140,141,142,143,144,145]. Besides some exceptions, electrochemistry is the preferred approach in MN-based biosensor design owing to many notable properties, including inherent miniaturization, highly scalable fabrication, rapid, inexpensive, low-power consumption requirements, and easier deployment to MNs [36,146,147,148,149,150]. Various surface chemistry procedures and transduction modes can be combined and deployed into MN-based biosensors according to the selection of targeted biomolecule. The great potential of MNs to enhance sensing ability for different targets in the detection of CDs has drawn much attention from researchers [151]. Despite the advantages, the application of MNs in detecting CD-related biomarkers is considerably new and a very limited number of applications has been reported. In the following sub-sections, we overview these applications of MNs in the diagnosis of different diseases’ biomarkers of CDs.

### 3.1. Diabetes Mellitus

**Figure 1:**
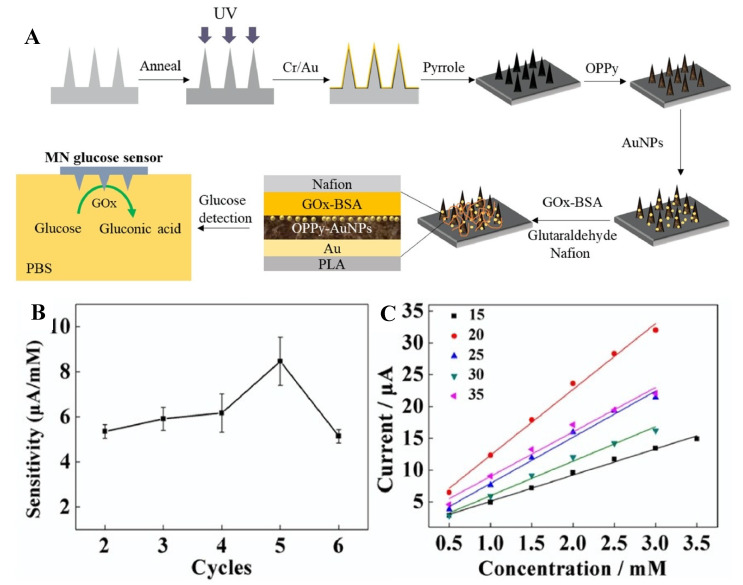
(**A**) The fabrication process of Nafion/GOx/AuNPs/overoxidized polypyrrole/Au MN array glucose biosensor. (**B**) The sensitivity curves of glucose biosensors with different cycles. (**C**) The calibration curves of glucose biosensors with different concentrations. Reprinted with permission from ref. [153] Copyright 2020, Elsevier.

Diabetes mellitus is an incurable disease caused by insulin deficiency in the body, resulting in hyperglycemia or hypoglycemia [152]. Monitoring glucose level is critical in managing this disease. In this context, a polylactic acid-based MN glucose biosensor has been developed [153]. This MN was then converted to a conductive platform by coating with a gold layer, after overoxidized polypyrrole, gold nanoparticles (AuNPs), glucose oxidase (GOx), and Nafion as shown in Figure 1A. This glucose MN biosensor was reported to show a 40 μM limit of detection (LOD) with a sensitivity of 8.09 μA/mM and a linear range of 0 to 2.6 mM in vitro tests (Figure 1B,C). The combination of overoxidized polypyrrole and AuNPs increased the sensor’s sensitivity and facilitated the adhesion of GOx to the electrode, while the Nafion film protected the sensor from intrusive substances. This developed biosensor maintained its stability after 14 days by exhibiting a constant tracking sensitivity.

In another study, a biocompatible and invasive hollow MN platform integrated into a paper-based multiplex biosensor was designed to measure glucose and cholesterol levels in patients in multiple ways [141]. After blood sampling, which can be performed painlessly with minimal tissue damage and inflammation through hollow MN, separation and detection are sequentially integrated and automated in a single device without any additional processes. This device can also perform blood sampling and measure glucose and cholesterol levels entirely automatically. A glucose-responsive colloidal crystal MN patch is presented in a recent study, aiming to monitor glucose levels with a naked eye [154]. In order to convert physiological glucose levels into color signals, it was formed with a colloidal crystal, SiO_2_ nanoparticles, and a glucose-sensitive fluorophenylboronic acid-based matrix. These glucose-sensitive colloidal crystals were coated onto the polymerized MN surface through a secondary photocrosslinking method, which was mediated by residual double bonds on the MN. The resulting core-shell structure not only preserves the sensitivity of colloidal crystals, but also ensures that the MN has a suitable skin penetration feature. The MN patch reversibly converts glucose concentrations to color changes within 5 min that can be monitored by a naked eye.

The demand for user-friendly and functional biosensor systems for the simultaneous detection of multiple blood metabolites has recently increased, especially for home-based health care and monitoring strategies. Based on this demand, a flexible MN electrode array-based biosensor and a portable multichannel electrochemical analyzer have been developed for the simultaneous detection of glucose, cholesterol, and uric acid [155]. Here, the MN electrode array was fabricated on a flexible substrate, and then, coated with gold/titanium film and biofunctionalized with glucose oxidase, cholesterol oxidase, and uricase in an order. This biosensor exhibited a detection capability ranging from 2 to 12 mM for glucose, from 1 to 12 mM for cholesterol, and from 0.1 to 1.2 mM for uric acid. The platform was also tested in serum samples, and provided detection limits of 260 μM for glucose, 440 μM for cholesterol, and 4 μM for uric acid. In sum, the facile fashion of this platform would hold a notable impact to accelerate the monitoring of blood metabolites effectively at the PoC settings.

**Figure 2:**
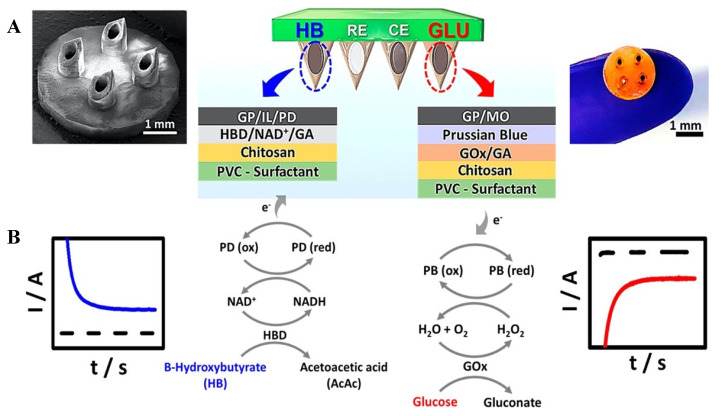
(**A**) The schematic representation of dual-marker β−hydroxybutyrate/glucose−sensing on a MN biosensor platform. (**B**) A schematic illustration of the dual-analyte amperometric detection mechanism on multilayer modified biosensors for β−hydroxybutyrate (marker) (**left**) and glucose (**right**). Reprinted with permission from ref. [157]. Copyright 2020, ACS Publications.

Diabetic ketoacidosis―a serious complication of diabetes, is characterized by hyperglycemia and metabolic acidosis due to the accumulation of ketone bodies. It should be followed along with glucose levels in diabetic patients [156]. In a study based on the absence of this monitoring system, an MN platform for detecting ketone bodies in real-time is presented [157]. This system is based on electrochemically monitoring β-hydroxybutyrate, one of the main biomarkers of ketone formation. Such real-time β-hydroxybutyrate detection addressed the main challenges related to the stable restriction of the enzyme/cofactor (NAD^+^) pair using the enzyme β-hydroxybutyrate dehydrogenase, and it was able to perform anodic detection of NADH (Figure 2A). This MN platform was able to detect β -hydroxybutyrate with low levels (LOD; 50 μM). Moreover, this MN biosensor was combined with an oxidase-based glucose biosensor on the same array platform, and a mechanism has been tailored to simultaneous monitoring of these diabetes markers (Figure 2B).

### 3.2. Cancer Monitoring

Cancer has a growing impact on the world population and the health care system globally. Cancer is considered a CD by the WHO and Centers for Disease Control and Prevention since most of the cancer survivors require ongoing treatment for several years [2,158]. Early detection of cancer is the mainstay for improving its prognosis, however, it still remains a challenge [159]. Cancer-related biomarkers are biological molecules that reveal the presence of cancer in a patient. The identification of such molecules is essential in the diagnosis and clinical management of the disease [160]. In this manner, MNs are proven to be a promising platform for the early detection and continuous monitoring of cancer biomarkers. As an example, for breast cancer, the tissue fluid of the breast provides critical information about the development progress of cancer (Stage I–V). Carcino-embryonic antigen (CEA), for instance, is one of the tumor biomarkers used for cancer diagnosis. In response to the given information, MNs were coated with Ag_3_PO_4_/Ag nanocomposite materials and MBL-modified pectin film for the biosensing of breast cancer [31]. Ag_3_PO_4_/Ag nanocomposite was utilized to facilitate the colorimetric sensing of the CEA in mice. MN-based sensing system had an excellent sensitivity to detect CEA at the concentration values varying from 0.2 ng/mL to 2.5 ng/mL. According to the preclinical experiments on humans (7 female patients, 3 healthy females), the MN-based system achieved to detect CEA biomarker in patients who have been diagnosed with breast cancer.

Moreover, nitric oxide (NO) has been found to inhibit the progression of cancer by decreasing tumor growth, and it is another important biomarker to monitor cancer progression. In this manner, for the first time, the MN biosensor system was fabricated using polycaprolactone (PCL) and integrated into the endomicroscopic system [161]. Poly (3,4-ethylenedioxythiophene) (PEDOT) was utilized to coat the MNs, which were further functionalized with hemin molecules to facilitate imaging and biosensing of colon cancer. The sensor was highly sensitive to the current change, and there was a significant difference in electrical read-out acquired from normal and cancer tissue in mice models. The LOD value was 1 × 10^−6^ M for NO sensing. In addition, this system showed great stability for several days.

Vascular endothelial growth factor is another important biomarker to monitor as it plays a critical role in controlling the growth and permeability of blood vessels [162]. Recent studies demonstrated an efficient functionalization method of MNs using peptide aptamer to detect electrochemical reactions with VEGF as a real-time cancer diagnostic platform. Functionalized MN-based biosensing system was capable of detecting VEGF in human blood serum with a detection limit of 0.1 to 1000 pM, showing a high VEGF selectivity.

**Figure 3:**
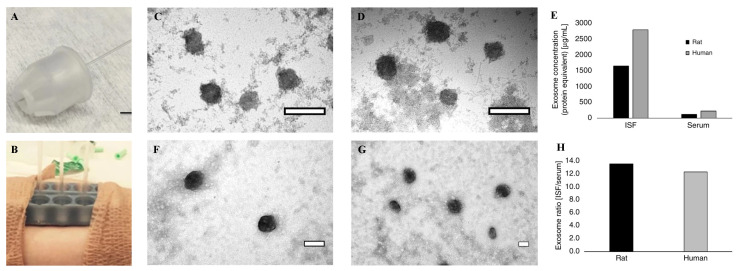
(**A**) The MN within polymer housing with glass capillary collection tube. (**B**) Two 3D MN holders were adhered to a human subject for interstitial fluid extraction and collection in capillary glass tubes. TEM images of rat (**C**,**D**) and human (**F**,**G**) exosomes purified from ISF (**C**,**F**) and serum (**D**,**G**) were exhibited. (**E**) Exosome concentration (protein equivalent) was measured using a NanoDrop system. (**H**) The exosome ratio of ISF vs. serum in rat and human samples were presented. Reprinted with permission from ref. [168].Copyright 2018, Nature.

Exosomes, on the other hand, are cell-derived vesicles, containing high concentrations of proteins, nucleic acids [163], and clinically essential biomarkers for cancer [164] and many CDs [165,166,167]. In a recent study, a hollow MN array was developed to extract large volumes (20–60 µL) of dermal interstitial fluid without causing any damage to the skin (Figure 3A,B) [168]. MNs were formed from 4 mm ultra-thin pen needles and the protective plastic housing was cut using a laser cutter. When the housing was pressed against the skin, it generated a concentric opening around the MN. Then, the interstitial fluid collection was performed with a glass capillary attached to the back of the MN, and the interstitial fluid entered the capillary within 10–15 min on average. After removal from the MN, the interstitial fluid collected in the capillary was colorless, and did not contain any red blood cells. Exosomes were then isolated from human interstitial fluid and serum successfully, as well as visualized via transmission electron microscope (TEM) (Figure 3C–G). With this MN platform, sufficient volumes of dermal interstitial fluid could be extracted for analysis, such as transcriptomic and proteomic profiling, and exosome isolation.

### 3.3. Chronic Kidney Disease

Chronic kidney disease (CKD) is a syndrome defined by changes in the kidney structure such as cysts, tumors, malformations, and atrophy. Furthermore, CKD has permanent effects such as edema in the kidney and changes in urine quality. These changes are usually described by increased levels of creatinine, cystatin C in serum, or urea nitrogen levels in blood [169]. In a study for the determination of urea, one of these biomarkers, a gold MN array was presented by casting conductive gold ink [170]. These MN arrays were functionalized with epoxy and ferrocene functional polymeric mediators and covalently immobilized urease for the use as an electrochemical biosensor. For the detection of urea, urea spiked artificial interstitial fluid was employed, and this biosensor was tested on artificial epidermal skin. According to the results obtained, this biosensor was able to detect urea within the range spanning from 50 to 2500 µM along with a LOD of 2.8 µM and sensitivity of 31 nA/mM. For the direct detection of cystatin C, another crucial biomarker of CKD, a carbohydrate MN device integrated with localized surface plasmon resonance (LSPR), has been developed [142]. These MNs were produced with maltose, and then, coated with poly lactic-co-glycolic acid. Because of the porous structure of poly lactic-co-glycolic acid, blood could be easily withdrawn by capillary action, as well as all blood was filtered and only plasma reached the bio-recognition layer. This biocompatible device was able to directly detect cystatin C in blood taken by finger-pricking at concentrations up to 0.01 μg/mL in buffered conditions.

### 3.4. Parkinson Disease

**Figure 4:**
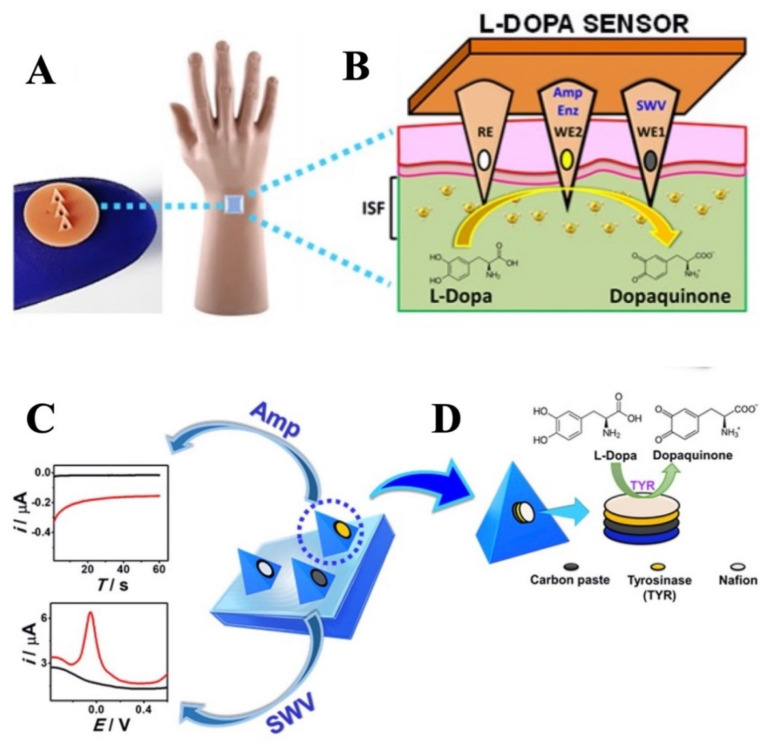
The schematic represents (**A**) the MN sensor and (**B**) the detection of L−Dopa in interstitial fluid. (**C**) The schematic illustration of the MN sensor platform depicts three electrodes for sensing Levodopa l through square-wave voltammetry and amperometry modalities. (**D**) MN sensor includes the corresponding reagent layers, i.e., the carbon paste, tyrosinase, and Nafion layer. Reprinted with permission from ref. [173]. Copyright 2019, ACS Publications.

Parkinson’s disease (PD) is a chronic neurodegenerative disorder that results from a synaptic and neuronal loss due to the misfolding of α-synuclein (αSyn)―the major protein marker [171]. Its clinical picture includes the main motor symptoms, such as rigidity, tremors, bradykinesia, and postural instability [172]. Moreover, Levodopa is one of the most effective drugs in the treatment of Parkinson’s disease. The management of Parkinson’s disease is however very difficult as the dose optimization of the drug is currently determined based on the symptom reports of patients, who are difficult to identify. For instance, in a recent study, an MN detection platform has been developed to continuously monitor Levodopa levels by employing electrochemical sensing modality [173]. This MN detection platform provided a multimodal sensing strategy through simultaneous enzymatic (amperometric) and non-enzymatic (voltammetric) detection of Levodopa using different MNs on the same biosensor array patch. For this purpose, square wave voltammetry and chronoamperometry were used in unmodified and tyrosinase modified carbon paste MN electrodes (Figure 4). This MN platform, allowing the detection of Levodopa in artificial interstitial fluid with high sensitivity and low LOD (~0.5 μM), has been an alternative method for the effective management of Parkinson’s disease and monitoring of Levodopa.

### 3.5. Chronic Electrolyte Disorders

Electrolytes are pivotal elements for cell signaling [174], homeostasis [175], and kidney function [176]. They also play a vital role in the regulation of CDs, such as cardiovascular diseases. For instance, the reduction in the concentration of certain electrolytes (i.e., sodium) in the body may result in chronic conditions, such as hyponatremia. Hence, their levels need to be constantly monitored. For the first time, an ion-selective transdermal MN sensor for potassium was designed by integrating a microfluidic chip with hollow micro-needle to eject fluid down a channel towards the solid-state ion-selective electrode [177]. The 3D porous carbon and 3D porous graphene electrodes formed by interference lithography were integrated for electrochemical measurements, and the performance of this design was evaluated in terms of stability and selectivity. The porous carbon electrodes performed better electrochemical performance (detection range from 10^−5^ to 10^−2^ M with a near Nernstian slope of 57.9 mV and rapid stabilization (≈20 s)) than those of the porous graphene electrodes. They were able to measure potassium at normal physiological concentrations, spanning from 10^–5^ to 10^–2^ M. On the other hand, the porous graphene electrode-integrated MN showed instability during this operation and measurements.

Moreover, skin interstitial fluids are rich sources of different types of electrolytes and their concentration is highly correlated with those in plasma. In a recent study, a MN-based potentiometric sensing system for continuous monitoring of Na^+^ and K^+^ in skin interstitial fluid was developed using micromilling machine [178]. The hollow MN was supported with polydimethylsiloxane (PDMS) and selective electrodes, as well as the reference electrode was inserted into the MN. The MN platform was able to detect both electrolytes below 15 mM. The hollow MN-based biosensor was also tested in artificial interstitial fluids and on chicken skin. The biosensor was both mechanically and chemically stable for 30 days, offering long-term monitoring of electrolytes. Other than electrolyte, the designed MNs were also selective toward metabolites (creatine and uric acid), nutrients (glutamine and vitamin A), and dietary biomarkers (caffeine) with a rapid turnaround and high repeatability.

## 4. Microneedles in Clinical Trials for CD Monitoring

Currently, approximately 62 clinical trials using MN technology have been reported on the ClinicalTrials.gov database [179]. The use of MNs in clinical trials is considerably new and more studies are required to encourage their use in clinics. Most of the reported clinical trials involving the use of MN technology is to evaluate their drug delivery efficiency for the treatment of different conditions. A very limited number of clinical trials have been published for the diagnosis and monitoring purpose of CDs.

According to the data obtained from clinicaltrials.gov, one of the clinical studies (ClinicalTrials.gov Identifier: NCT02682056) was first posted on 15 February 2016, involving MN patches for continuous monitoring of glucose in diabetic patients. This study was conducted with fifteen children and adolescents (7 years to 18 years) with diabetes, enrolled between December 2017 and January 2018. The detection efficiency of the MN patch, which was made from biocompatible polymers and metals, was compared to the samples collected using a lancet and an intravenous catheter. MN patch was designed to collect glucose from interstitial fluid while lancet and intravenous catheter detected the glucose collected from capillary blood glucose and venous glucose, respectively. The samples were collected hourly from all three devices for four hours. According to the primary outcome, three methods showed similar detection efficiency. The pain level assessment by pain Visual Analog Scale (0 indicating no pain and 100 indicating the most extreme pain) showed that the MN patches, lancet and intravenous catheter caused pain levels of 11.2, 4.4, and 26.8, respectively. In another pilot clinical study with both healthy volunteers (*n* = 8) and patients with Type 1 diabetes (*n* = 10), solid MN array-based biosensors were developed using polycarbonate MN arrays with a 1 mm thick base for monitoring glucose levels in real-time [146]. According to the results, the MN-based biosensor was highly tolerable compared to cannula insertion. The results acquired from amperometric biosensors confirmed the fact that glucose measurement performance was clinically acceptable with minimal discomfort and no inflammation.

Different studies have previously reported that there is a direct correlation between the use of antibiotics and CDs [180]. Hence, the continuous monitoring of antibiotics in patients with chronic conditions can be vital. An MN array platform (ClinicalTrials.gov Identifier: NCT03847610) was fabricated to continuously monitor β-Lactam antibiotic concentrations in a clinical trial with 11 healthy volunteers [181]. The MN platform was made of a poly (carbonate) base-coated with chrome and gold. Then, the base was coated with an insulating lacquer so that only the MNs possess conducting properties. Finally, MNs were deposited with a hydrogel containing β-lactamase. The sensing arrays consisted of three working electrodes functionalized with hydrogel and a reference electrode. The MN-based sensing system presented a good sensitivity, however the limit of detection of the sensor needed more improvements.

On the other hand, there are some recently completed and/orongoing clinical studies under this topic. For instance, a clinical trial (ClinicalTrials.gov Identifier: NCT03795402) using an MN device was conducted in March 2019 to collect and analyze samples for transcriptomics profiling from patients (*n* = 11) with mild chronic plaque Psoriasis Vulgaris which is a skin disorder. The study completion date is reported to be in December 2019; however, no results have been posted yet on the outcome of this clinical trial. According to the Asthma and Allergy Foundation of America (AAFA), allergies are one of the most common CDs. MNs were tested in a clinical trial (Clinical Trial Gov ID NCT01628484) on 20 participants (from 18 years to 65 years) with positive clinical history for inhalant allergy caused by birch pollen. The objective of this study was to determine allergic skin activity. Each patient was treated with three skin preparation techniques: (1) MNs, (2) Prick Lancet, and (3) tape stripping (Tesafilm). Unfortunately, no result is available on the results of this clinical trial since the last update in 2012.

## 5. Conclusions and Future Perspectives

Stating our personal views in this context, real-time monitoring of CDs still remains a challenge. Essentially, the development of effective biosensing devices is crucial to overcome these limitations associated with the current PoC platforms. Out of different approaches, MN-based platforms have been proven to significantly improve the detection efficiency. Recently, a great variety of MN-based biosensing platforms have been introduced. Currently, MN technologies still face certain obstacles. One field that still needs attention is regarding the efficacy of in vivo sampling with MN-based biosensors. Unfortunately, to date, only a few clinical trials involving such sensors have been reported. Certain strategies need to be followed to accelerate the translation of MN-based platforms from preclinical development into clinical trials.

One of the strategies is to propose easier and more affordable microfabrication techniques using more accessible materials to manufacture MNs. Advancements in ultra-rapid prototyping techniques would allow high-throughput manufacturing of MNs. For instance, one of the promising manufacturing technologies would be 3D printing. Recent breakthroughs in engineering inspired by the progress of photopolymerization techniques using a variety of material have made the manufacturing of such MNs with desired structures possible [182]. However, particular attention needs to be drawn to the fused deposition modeling and photopolymerization (i.e., two-photon photopolymerization). Additionally, expanding the range of printing materials would also be of great advantage to facilitate the manufacturing process. Other prototyping techniques (i.e., precision micro-milling or wire electrical discharge machining) would also help high-throughput manufacturing of MNs to be used for biosensor purpose. Furthermore, ongoing discovery of new biomarkers associated with CD needs to be integrated with recent technological advances in order to design effective MNs for the detection of such biomarkers. Nevertheless, more investigations are required to explore the molecular basis of CDs for the discovery of novel biomarkers. The designed MN-based biosensor needs to monitor multiple analytes at considerably low concentrations in situ without requirement of any pre-treatment or pre-processing steps. Hence, the enhancement of sensitivity, as well as selectivity of the MN is the primary concern. This can be achieved by conductively treating the surface of the MNs for electrical recording or grafting the surface of the MN with pH or light-responsive materials. With such surface functionalization, a great variety of diseases such as ALS (Lou Gehrig’s Disease), cystic fibrosis, and Dementia would be easily monitored.

In a nutshell, the MN-based biosensor would be integrated both in the process of acquiring time-sensitive and clinically valuable information through sensing and/or in long-term wearable monitoring systems. Consequently, the advances in MN-based biosensors for medical applications for detecting CDs would contribute to global healthcare, as well as potentially reducing healthcare disparities, regardless of the socio-economic group, with advancements in large-scale and inexpensive manufacturing of these platforms. In due course, it is expected that new advances in MN technology would accelerate to have the complete control of disease progression.

## Data Availability

Not applicable.
